# Multimodal 4-arylchromene derivatives with microtubule-destabilizing, anti-angiogenic, and MYB-inhibitory activities

**DOI:** 10.20517/cdr.2022.90

**Published:** 2023-02-01

**Authors:** Leonhard H. F. Köhler, Sebastian Reich, Maria Yusenko, Karl-Heinz Klempnauer, Gerrit Begemann, Rainer Schobert, Bernhard Biersack

**Affiliations:** ^1^Organic Chemistry Laboratory, University of Bayreuth, Bayreuth 95440, Germany.; ^2^Institute for Biochemistry, Westfälische-Wilhelms-Universität, Münster 48149, Germany.; ^3^Developmental Biology, University of Bayreuth, Bayreuth 95440, Germany.

**Keywords:** Chromene, pyran, anticancer drugs, microtubule, angiogenesis, MYB inhibition

## Abstract

**Aim:** Efficient and readily available anticancer drugs are sought as treatment options. For this reason, chromene derivatives were prepared using the one-pot reaction and tested for their anticancer and anti-angiogenic properties.

**Methods:** 2-Amino-3-cyano-4-(aryl)-7-methoxy-4H-chromene compounds (2A-R) were repurposed or newly synthesized via a three-component reaction of 3-methoxyphenol, various aryl aldehydes, and malononitrile. We performed assays to study the inhibition of tumor cell growth [3-(4, 5-dimethylthiazol-2-yl)-2, 5-diphenyl tetrazolium bromid (MTT) assay], effects on microtubules (immunofluorescence), cell cycle (flow-activated cell sorting analysis), angiogenesis (zebrafish model), and MYB activity (luciferase reporter assay). Fluorescence microscopy was applied for localization studies via copper-catalyzed azide-alkyne click reaction of an alkyne-tagged drug derivative.

**Results:** Compounds 2A-C and 2F exhibited robust antiproliferative activities against several human cancer cell lines (50% inhibitory concentrations in the low nanomolar range) and showed potent MYB inhibition. The alkyne derivative 3 was localized in the cytoplasm after only 10 min of incubation. Substantial microtubule disruption and G2/M cell-cycle arrest were observed, where compound 2F stood out as a promising microtubule-disrupting agent. The study of anti-angiogenic properties showed that 2A was the only candidate with a high potential to inhibit blood vessel formation *in vivo*.

**Conclusion:** The close interplay of various mechanisms, including cell-cycle arrest, MYB inhibition, and anti-angiogenic activity, led to identifying promising multimodal anticancer drug candidates.

## INTRODUCTION

**Figure 1:**
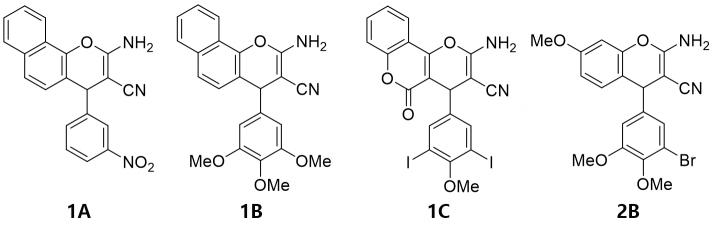
Structures of LY290181 (1A); MYB inhibitor Bcr-TMP (1B); chromosomal de-clustering agent 1C; and antiproliferative compound 2B.

Multi-component reactions such as the Biginelli reaction, Van Leusen reaction, and the Ugi reaction, and their modifications are helpful for the design of biologically active compounds^[1-4]^. An efficient three-component synthesis of biologically active pyrans was reported using naphthol, phenol, and hydroxyquinoline derivatives, which led to several new anticancer compounds^[5-8]^. For instance, the naphthopyran LY290181 or 2-amino-4-(3-nitrophenyl)-4*H*-naphtho(1, 2-*b*)pyran-3-carbonitrile (1A) was identified as an active anticancer MDA (microtubule-binding/destabilizing agent)^[9,10]^. The close analog 1B was initially described as an apoptosis inducer in breast cancer and non-small-cell lung cancer cells^[6]^. It was also recently determined to be a highly potent inhibitor of the transcription factor MYB^[11]^. MYB is encoded by the proto-oncogene MYB and is involved in the development of malignancies, making it a potential therapeutic target^[12]^. The structurally optimized 7-methoxy-4*H*-chromene 2B is an even more potent apoptosis-inducing agent than 1B and showed high antiproliferative activities^[5]^. The 2-amino-4-aryl-5-oxo-4, 5-dihydropyrano[3,2-c]chromene-3-carbonitrile 1C inhibited centrosome clustering in melanoma cells, leading to the formation of multiple mitotic spindles, chromosome segregation defects, and cell death [Figure 1]^[13]^.

Based on these active anticancer pyran heterocycles, several 2-amino-3-cyano-4-aryl-7-methoxy-4*H*-chromenes were prepared for testing their antiproliferative and MYB-inhibitory activities. Experiments on cell death induction, microtubule destabilization, drug localization, and anti-angiogenic effects were performed to investigate the underlying mechanisms of action of the most active compounds.

## METHODS

### Chemistry

#### General

Starting compounds and reagents were obtained from Aldrich, TCI, and Alfa Aesar. The already published compounds 1A, 1B, 2B, 2O, and 2R were prepared as described^[5,6,9,14]^. For compound analysis, the following instruments were used: Gallenkamp for melting points (uncorrected); Perkin-Elmer Spectrum One FT-IR spectrophotometer (ATR) for IR spectra; BRUKER Avance 300 spectrometer for nuclear magnetic resonance spectra; chemical shifts were expressed as ppm (parts per million, δ) downfield from TMS (tetramethylsilane) as the internal standard; Varian MAT 311A (EI) and UPLC/Orbitrap (ESI-HRMS) for mass spectra; Perkin-Elmer 2400 CHN elemental analyzer for elemental analyses (microanalyses), and compounds were > 95% pure as to elemental analysis.

#### Synthesis

2-Amino-3-cyano-4-(3’-chloro-4’, 5’-dimethoxyphenyl)-7-methoxy-4H-chromene (2A)

3-Chloro-4, 5-dimethoxybenzaldehyde (200 mg, 1.0 mmol) and malononitrile (70 mg, 1.0 mmol) were dissolved in MeCN (5 mL), and three drops of Et_3_N were added. The reaction mixture was stirred at room temperature for 30 min. 3-Methoxyphenol (109 µL, 1.0 mmol) was added, and the reaction mixture was heated with a heat gun and stirred at room temperature for 2 h. The precipitate was collected, washed with MeCN and *n*-hexane, and dried in a vacuum. Yield: 80 mg (0.22 mmol, 22%); colorless solid of mp = 224-225 °C;* υ*_max_(ATR)/cm^-1^ 3397, 3332, 3220, 2943, 2846, 2186, 1651, 1619, 1572, 1509, 1465, 1430, 1415, 1399, 1313, 1281, 1258, 1231, 1207, 1193, 1178, 1148, 1137, 1124, 1051, 998, 928, 856, 834, 813, 801, 780, 753, 714, 690, 659; ^1^H NMR (300 MHz, CDCl_3_) 3.77 (3 H, s, OMe), 3.82 (6 H, s, 2 x OMe), 4.5-4.6 (3 H, m, 4-H, NH_2_), 6.53 (1 H, d, J = 2.5 Hz, 8-H), 6.6-6.7 (3 H, m, 6’-H, 6-H), 6.71 (1 H, s, 2’-H), 6.85 (1 H, d, J = 8.7 Hz, 5-H); ^13^C NMR (75.5 MHz, CDCl_3_) 40.2 (4-C), 55.5 (OMe), 56.2 (OMe), 60.7 (OMe), 101.4 (8-C), 110.6 (6-C), 111.8 (6’-C), 113.9 (4a-C_q_), 119.6 (CN), 121.2 (2’-C), 128.5 (3’-C), 130.1 (1’-C), 141.3 (4’-C), 149.1 (5’-C), 153.9 (8a-C_q_), 159.1 (7-C), 159.6 (2-C); HRMS for C_19_H_18_O_4_N_2_Cl [M^+^ + H] calcd. 373.09496, found 373.09355.

2-Amino-3-cyano-4-(3’-iodo-4’, 5’-dimethoxyphenyl)-7-methoxy-4H-chromene (2C)

Analogously to the synthesis of 2B, compound 2C (150 mg, 0.32 mmol, 32%) was obtained from 3-iodo-4, 5-dimethoxybenzaldehyde (292 mg, 1.0 mmol), malononitrile (70 mg, 1.0 mmol), three drops of Et_3_N, and 3-methoxyphenol (109 µL, 1.0 mmol) in MeCN (5 mL). Colorless solid of m.p. 223-224 °C;* υ*_max_(ATR)/cm^-1^ 3403, 3334, 3220, 2937, 2841, 2186, 1651, 1619, 1582, 1563, 1508, 1479, 1464, 1411, 1397, 1308, 1272, 1253, 1231, 1200, 1178, 1136, 1124, 1044, 999, 875, 855, 812, 801, 777, 750, 688, 655, 611;^ 1^H NMR (300 MHz, dimethyl sulfoxide [DMSO]-d_6_) 3.67 (3 H, s, OMe), 3.74 (3 H, s, OMe), 3.78 (3 H, s, OMe), 4.68 (1 H, s, 4-H), 6.56 (1 H, d, J = 2.5 Hz, 8-H), 6.68 (1 H, dd, J = 8.7 Hz, 2.5 Hz, 6-H), 6.9-7.1 (5 H, m, 6’-H, 6-H, 5-H, NH_2_); ^13^C NMR (75.5 MHz, DMSO-d_6_) 39.1 (4-C), 55.4 (OMe), 55.6 (OMe), 55.9 (OMe), 93.0 (3’-C), 100.9 (8-C), 111.4 (6-C), 112.7 (6’-C), 114.8 (4a-C_q_), 120.4 (CN), 128.2 (5-C), 129.9 (2’-C), 144.4 (1’-C), 146.9 (4’-C), 148.7 (5’-C), 152.2 (8a-C_q_), 159.0 (7-C), 160.3 (2-C);* m/z* (%) 464 (43) [M^+^], 201 (100); Anal. calcd. for C_19_H_17_IN_2_O_4_ (%), C, 49.16, H, 3.69, N, 6.03; found: C, 49.24, H, 3.77, N, 5.98.

2-Amino-3-cyano-4-(3’, 5’-dichloro-4’-methoxyphenyl)-7-methoxy-4H-chromene (2D)

Analogously to the synthesis of 2B, compound 2D (115 mg, 0.31 mmol, 31%) was obtained from 3,5-dichloro-4-methoxybenzaldehyde (205 mg, 1.0 mmol), malononitrile (70 mg, 1.0 mmol), three drops of Et_3_N, and 3-methoxyphenol (109 µL, 1.0 mmol) in MeCN (5 mL). Colorless solid of m.p. 229 °C;* υ*_max_(ATR)/cm^-1^ 3395, 3331, 3219, 3004, 2973, 2938, 2841, 2192, 1652, 1619, 1583, 1557, 1509, 1478, 1448, 1404, 1395, 1318, 1301, 1282, 1265, 1254, 1195, 1150, 1127, 1082, 1043, 1029, 996, 949, 914, 888, 857, 848, 807, 795, 775, 751, 741, 687, 667, 653; ^1^H NMR (300 MHz, CDCl_3_/DMSO-d_6_) 3.62 (3 H, s, OMe), 3.70 (3 H, s, OMe), 4.44 (1 H, s, 4-H), 5.58 (2 H, s, NH_2_), 6.4-6.5 (2 H, m, 6-H, 8-H), 6.6-6.7 (1 H, m, 5-H), 6.96 (2 H, s, 2’-H, 6’-H); ^13^C NMR (75.5 MHz, CDCl_3_/DMSO-d_6_) 39.7 (4-C), 55.4 (OMe), 57.7 (3-C), 60.5 (OMe), 101.4 (8-C), 111.5 (6-C), 113.5 (4a-C_q_), 120.2 (CN), 128.0 (2’-C, 6’-C), 129.3 (3’-C, 5’-C), 129.9 (5-C), 142.9 (1’-C), 149.0 (4’-C), 150.9 (8a-C_q_), 159.5 (7-C), 160.0 (2-C); *m/z* (EI) 378 (16) [M^+^], 376 (23) [M^+^], 201 (100); Anal. calcd. for C_18_H_14_Cl_2_N_2_O_3_ (%), C, 57.31, H, 3.74, N, 7.43; found: C, 57.40, H, 3.70, N, 7.36.

2-Amino-3-cyano-4-(3’, 5’-dibromo-4’-methoxyphenyl)-7-methoxy-4H-chromene (2E)

Analogously to the synthesis of 2B, compound 2E (125 mg, 0.27 mmol, 31%) was obtained from 3, 5-dibromo-4-methoxybenzaldehyde (259 mg, 0.88 mmol), malononitrile (70 mg, 1.0 mmol), three drops of Et_3_N, and 3-methoxyphenol (109 µL, 1.0 mmol) in MeCN (5 mL). Colorless solid of m.p. 247 °C;* υ*_max_(ATR)/cm^-1^ 3396, 3331, 3218, 2943, 2190, 1652, 1619, 1582, 1547, 1508, 1471, 1420, 1407, 1394, 1317, 1296, 1282, 1253, 1194, 1150, 1125, 1064, 1045, 1028, 999, 990, 947, 888, 857, 847, 811, 800, 779, 750, 733, 704, 686, 663; ^1^H NMR (300 MHz, CDCl_3_/DMSO-d_6_) 3.70 (3 H, s, OMe), 3.76 (3 H, s, OMe), 4.52 (1 H, s, 4-H), 5.90 (2 H, s, NH_2_), 6.47 (1 H, d, J = 2.5 Hz, 8-H), 6.55 (1 H, dd, J = 8.6 Hz, 2.5 Hz, 6-H), 6.77 (1 H, d, J = 8.6 Hz, 5-H), 7.23 (2 H, s, 2’-H, 6’-H); ^13^C NMR (75.5 MHz, CDCl_3_/DMSO-d_6_) 39.1 (4-C), 54.9 (OMe), 56.9 (3-C), 59.9 (OMe), 101.0 (8-H), 111.0 (6-C), 113.1 (4a-C_q_), 117.6 (3’-C, 5’-C), 119.7 (CN), 129.4 (5-C), 131.3 (2’-C, 6’-C), 143.7 (1’-C), 148.6 (4’-C), 152.2 (8a-C_q_), 159.0 (7-C), 159.7 (2-C); *m/z* (EI) 468 (8) [M^+^], 466 (16) [M^+^], 464 (8) [M^+^], 201 (100); Anal. calcd. for C_18_H_14_Br_2_N_2_O_3_ (%), C, 46.38, H, 3.03, N, 6.01; found: C, 46.29, H, 2.98, N, 6.06.

2-Amino-3-cyano-4-(3, 5-diiodo-4-methoxyphenyl)-7-methoxy-4H-chromene (2F)

Analogously to the synthesis of 2B, compound 2F (165 mg, 0.30 mmol, 30%) was obtained from 3, 5-diiodo-4-methoxybenzaldehyde (388 mg, 1.0 mmol), malononitrile (70 mg, 1.0 mmol), three drops of Et_3_N, and 3-methoxyphenol (109 µL, 1.0 mmol) in MeCN (5 mL). Colorless solid of m.p. 228 °C;* υ*_max_(ATR)/cm^-1^ 3400, 3332, 3219, 2936, 2841, 2187, 1652, 1619, 1580, 1534, 1506, 1460, 1405, 1387, 1316, 1293, 1281, 1247, 1198, 1150, 1124, 1048, 1028, 993, 945, 897, 885, 854, 808, 798, 778, 749, 737, 711, 708, 700, 685; ^1^H NMR (300 MHz, CDCl_3_/DMSO-d_6_) 3.70 (3 H, s, OMe), 3.72 (3 H, s, OMe), 4.49 (1 H, s, 4-H), 6.07 (2 H, s, NH_2_), 6.47 (1 H, d, J = 2.6 Hz, 8-H), 6.54 (1 H, dd, J = 8.6 Hz, 2.6 Hz, 6-H), 6.77 (1 H, d, J = 8.6 Hz, 5-H), 7.47 (2 H, s, 2’-H, 6’-H); ^13^C NMR (75.5 MHz, CDCl_3_/DMSO-d_6_) 54.9 (OMe), 56.6 (3-C), 59.9 (OMe), 90.3 (2’-C, 6’-C), 100.9 (8-C), 111.0 (6-C), 113.3 (4a-C_q_), 119.8 (CN), 129.4 (5-C), 138.4 (2’-C, 6’-C), 144.9 (1’-C), 148.6 (8a-C_q_), 156.9 (4’-C), 158.9 (7-C), 159.7 (2-C); *m/z* (EI) 560 (52) [M^+^], 201 (100); Anal. calcd. for C_18_H_14_I_2_N_2_O_3_ (%), C, 38..60, H, 2.52, N, 5.00; found: C, 38.69, H, 2.45, N, 4.97.

2-Amino-3-cyano-4-(2’-fluorophenyl)-7-methoxy-4H-chromene (2G)

Analogously to the synthesis of 2B, compound 2G (73 mg, 0.25 mmol, 25%) was obtained from 2-fluorobenzaldehyde (124 mg, 1.0 mmol), malononitrile (70 mg, 1.0 mmol), three drops of Et_3_N, and 3-methoxyphenol (109 µL, 1.0 mmol) in MeCN (5 mL). Yield: 73 mg (0.25 mmol, 25%); colorless solid of m.p. 198 °C;* υ*_max_(ATR)/cm^-1^ 3405, 3336, 3214, 2194, 1652, 1619, 1582, 1509, 1486, 1453, 1435, 1405, 1317, 1292, 1255, 1240, 1224, 1193, 1152, 1124, 1103, 1092, 1045, 1031, 937, 871, 857, 817, 803, 787, 753, 721, 705, 679; ^1^H NMR (300 MHz, CDCl_3_) 3.75 (3 H, s, OMe), 4.65 (2 H, s, NH_2_), 5.04 (1 H, s, 4-H), 6.51 (1 H, d, J = 2.6 Hz, 8-H), 6.59 (1 H, dd, J = 8.6 Hz, 2.6 Hz, 6-H), 6.92 (1 H, d, J = 8.6 Hz, 5-H), 7.0-7.2 (4 H, m, 3’-H, 4’-H, 5’-H, 6’-H); ^13^C NMR (75.5 MHz, CDCl_3_) 33.8 (4-C), 55.5 (OMe), 59.2 (3-C), 101.3 (8-C), 111.7 (6-C), 114.1 (4a-C_q_), 115.8 (d, J = 22.0 Hz, 3’-C), 119.6 (CN), 124.6 (5’-C), 128.9 (d = 8.3 Hz, 4’-C), 129.7 (5-C), 131.5 (d = 12.7 Hz, 6’-C), 149.2 (8a-C_q_), 159.4 (7-C), 159.8 (2-C), 160.0 (d = 195.9 Hz, 2’-C); *m/z* (EI) 296 (42) [M^+^], 201 (100); Anal. calcd. for C_17_H_13_FN_2_O_2_ (%), C, 68.91, H, 4.42, N, 9.45; found: C, 68.99, H, 4.36, N, 9.39.

2-Amino-3-cyano-4-(2’-chlorophenyl)-7-methoxy-4H-chromene (2H)

Analogously to the synthesis of 2B, compound 2H (500 mg, 1.6 mmol, 40%) was obtained from 2-chlorobenzaldehyde (562 mg, 4.0 mmol), malononitrile (280 mg, 4.0 mmol), six drops of Et_3_N, and 3-methoxyphenol (436 µL, 4.0 mmol) in MeCN (5 mL). Colorless solid of m.p. 184 °C;* υ*_max_(ATR)/cm^-1^ 3435, 3343, 3216, 2937, 2840, 2186, 1639, 1618, 1580, 1506, 1466, 1441, 1402, 1311, 1291, 1278, 1253, 1198, 1154, 1123, 1033, 956, 935, 874, 854, 817, 782, 758, 724, 708, 694, 675; ^1^H NMR (300 MHz, CDCl_3_) 3.75 (3 H, s, OMe), 4.62 (2 H, s, NH_2_), 5.31 (1 H, s, 4-H), 6.51 (1 H, d, J = 2.6 Hz, 8-H), 6.57 (1 H, dd, J = 8.6 Hz, 2.6 Hz, 6-H), 6.89 (1 H, d, J = 8.6 Hz, 5-H), 7.2-7.3 (3 H, m, 4’-H, 5’-H, 6’-H), 7.3-7.4 (1 H, m, 3’-H); ^13^C NMR (75.5 MHz, CDCl_3_) 36.8 (3-C), 55.5 (OMe), 59.7 (4-C), 101.3 (8-H), 111.6 (6-C), 114.2 (4a-C_q_), 119.5 (CN), 127.5 (6’-C), 128.4 (5’-C), 129.7 (4’-C), 129.9 (3’-C), 130.7 (5-C), 133.0 (2’-C), 141.8 (1’-C), 149.1 (8a-C_q_), 159.5 (7-C), 159.6 (2-C); *m/z* (EI) 314 (6) [M^+^], 312 (24) [M^+^], 201 (100); Anal. calcd. for C_17_H_13_ClN_2_O_2_ (%), C, 65.29, H, 4.19, N, 8.96; found: C, 65.36, H, 4.12, N, 9.02.

2-Amino-3-cyano-4-(3’, 4’-difluorophenyl)-7-methoxy-4H-chromene (2I)

Analogously to the synthesis of 2B, compound 2I (98 mg, 0.31 mmol, 31%) was obtained from 3, 4-difluorobenzaldehyde (142 mg, 1.0 mmol), malononitrile (70 mg, 1.0 mmol) three drops of Et_3_N, and 3-methoxyphenol (109 µL, 1.0 mmol) in MeCN (5 mL). Yield: 98 mg (0.31 mmol, 31%); colorless solid of m.p. 193 °C;* υ*_max_(ATR)/cm^-1^ 3433, 3349, 3220, 2842, 2186, 1663, 1616, 1580, 1507, 1464, 1436, 1408, 1311, 1289, 1255, 1202, 1191, 1156, 1123, 1109, 1046, 959, 928, 889, 856, 828, 816, 802, 775, 746, 697, 673; ^1^H NMR (300 MHz, CDCl_3_) 3.77 (3 H, s, OMe), 4.61 (2 H, s, NH_2_), 4.64 (1 H, s, 4-H), 6.53 (1 H, d, J = 2.6 Hz, 8-H), 6.62 (1 H, dd, J = 8.6 Hz, 2.6 Hz, 6-H), 6.81 (1 H, d, J = 8.6 Hz, 5-H), 6.9-7.0 (2 H, m, 5’-H, 6’-H), 7.0-7.1 (1 H, m, 2’-H); ^13^C NMR (75.5 MHz, CDCl_3_) 39.8 (4-C), 55.5 (OMe), 60.5 (3-C), 101.5 (8-C), 111.9 (6-C), 113.8 (4a-C_q_), 116.7 (d, J = 17.6 Hz, 2’-C), 117.4 (d, J = 17.4 Hz, 5’-C), 119.4 (CN), 123.7 (d, J = 9.8 Hz, 6’-C), 130.1 (5-C), 141.8 (1’-C), 147.1 (d, J = 156.0 Hz), 149.1 (8a-C_q_), 151.2 (d, J = 156.0 Hz, 3’-C), 159.1 (7-C), 159.7 (2-C); *m/z* (EI) 314 (33) [M^+^], 201 (100); Anal. calcd. for C_17_H_12_F_2_N_2_O_2_ (%), C, 64.97, H, 3.85, N, 8.91; found: C, 65.08, H, 3.79, N, 8.96.

2-Amino-3-cyano-4-(3’-chloro-4’-fluorophenyl)-7-methoxy-4H-chromene (2J)

Analogously to the synthesis of 2B, compound 2J (100 mg, 0.30 mmol, 30%) was obtained from 3-chloro-4-fluorobenzaldehyde (159 mg, 1.0 mmol), malononitrile (70 mg, 1.0 mmol), three drops of Et_3_N, and 3-methoxyphenol (109 µL, 1.0 mmol) in MeCN (5 mL). Colorless solid of m.p. 192-193 °C.* υ*_max_(ATR)/cm^-1^ 3432, 3350, 3285, 3219, 3087, 2972, 2842, 2186, 1659, 1615, 1579, 1505, 1495, 1464, 1435, 1412, 1401, 1320, 1306, 1291, 1254, 1221, 1201, 1153, 1122, 1058, 1044, 948, 897, 855, 828, 815, 801, 774, 755, 711, 702, 693;^ 1^H NMR (300 MHz, CDCl_3_) 3.77 (3 H, s, OMe), 4.61 (2 H, s, NH_2_), 4.64 (1 H, s, 4-H), 6.54 (1 H, d, J = 2.6 Hz, 8-H), 6.61 (1 H, dd, J = 8.6 Hz, 2.6 Hz, 6-H), 6.80 (1 H, d, J = 8.6 Hz, 5-H), 7.0-7.1 (2 H, m, 5’-H, 6’-H), 7.1-7.2 (1 H, m, 2’-H); ^13^C NMR (75.5 MHz, CDCl_3_) 39.7 (4-C), 55.6 (OMe), 60.5 (3-C), 101.5 (8-C), 112.0 (6-C), 113.8 (4a-C_q_), 116.8 (d, J = 21.3 Hz, 5’-C), 119.4 (CN), 121.4 (d, J = 18.0 Hz, 3’-C), 127.6 (6’-C), 130.0 (2’-C), 131.1 (5-C), 141.9 (1’-C), 149.1 (8a-C_q_), 157.3 (d, J = 248.9 Hz, 4’-C), 159.1 (7-C), 159.7 (2-C);* m/z* (%) 330 (47) [M^+^], 201 (100), 186 (43), 158 (43). Anal. calcd. for C_17_H_12_ClFN_2_O_2_ (%), C, 61.74, H, 3.66, N, 8.47; found: C, 61.84, H, 3.71, N, 8.50.

2-Amino-3-cyano-4-(3’, 4’-dichlorophenyl)-7-methoxy-4H-chromene (2K)

Analogously to the synthesis of 2B, compound 2K (105 mg, 0.30 mmol, 30%) was obtained from 3, 4-dichlorobenzaldehyde (175 mg, 1.0 mmol), malononitrile (70 mg, 1.0 mmol), three drops of Et_3_N, and 3-methoxyphenol (109 µL, 1.0 mmol) in MeCN (5 mL). Colorless solid of m.p. 192 °C;* υ*_max_(ATR)/cm^-1^ 3433, 3349, 3220, 2186, 1661, 1616, 1579, 1506, 1463, 1434, 1412, 1320, 1290, 1251, 1199, 1180, 1157, 1123, 1046, 1029, 948, 911, 895, 885, 856, 825, 809, 786, 753, 732, 703, 692, 665; ^1^H NMR (300 MHz, CDCl_3_) 3.77 (3 H, s, OMe), 4.63 (1 H, s, 4-H), 4.66 (2 H, s, NH_2_), 6.53 (1 H, d, J = 2.6 Hz, 8-H), 6.61 (1 H, dd, J = 8.6 Hz, 2.6 Hz, 6-H), 6.80 (1 H, d, J = 8.6 Hz, 5-H), 7.03 (1 H, dd, J = 8.2 Hz, 2.1 Hz, 6’-H), 7.22 (1 H, d, J = 2.1 Hz, 2’-H), 7.37 (1 H, d, J = 8.2 Hz, 5’-H); ^13^C NMR (75.5 MHz, CDCl_3_) 39.8 (4-C), 55.5 (OMe), 60.0 (3-C), 101.5 (8-C), 111.9 (6-C), 113.5 (4a-C_q_), 119.4 (CN), 127.3 (6’-C), 129.8 (2’-C), 130.1 (5-C), 130.8 (5’-C), 131.4 (4’-C), 132.9 (3’-C), 145.0 (1’-C), 149.0 (8a-C_q_), 159.2 (7-C), 159.7 (2-C); *m/z* (EI) 348 (7) [M^+^], 346 (13) [M^+^], 201 (100); Anal. calcd. for C_17_H_12_Cl_2_N_2_O_2_ (%), C, 58.81, H, 3.48, N, 8.07; found: C, 58.94, H, 3.55, N, 8.09.

2-Amino-3-cyano-4-(2’, 4’-difluorophenyl)-7-methoxy-4H-chromene (2L)

Analogously to the synthesis of 2B, compound 2L (100 mg, 0.32 mmol, 32%) was obtained from 2, 4-difluorobenzaldehyde (142 mg, 1.0 mmol), malononitrile (70 mg, 1.0 mmol), three drops of Et_3_N, and 3-methoxyphenol (109 µL, 1.0 mmol) in MeCN (5 mL). Colorless solid of m.p. 169-170 °C.* υ*_max_(ATR)/cm^-1^ 3432, 3349, 3220, 3084, 3015, 2969, 2936, 2840, 2186, 1660, 1615, 1602, 1581, 1497, 1463, 1409, 1316, 1280, 1262, 1241, 1220, 1202, 1190, 1155, 1138, 1124, 1086, 1039, 960, 852, 827, 808, 781, 751, 730, 683; ^1^H NMR (300 MHz, CDCl_3_) 3.76 (3 H, s, OMe), 4.63 (2 H, s, NH_2_), 5.01 (1 H, s, 4-H), 6.52 (1 H, d, J = 2.6 Hz, 8-H), 6.60 (1 H, dd, J = 8.6 Hz, 2.6 Hz, 6-H), 6.7-6.8 (2 H, m, 3’-H, 5’-H), 6.88 (1 H, d, J = 8.6 Hz, 5-H), 7.0-7.1 (1 H, m, 6’-H); ^13^C NMR (75.5 MHz, CDCl_3_) 33.5 (4-C), 55.5 (OMe), 59.2 (3-C), 101.4 (8-C), 103.8-104.5 (m, 3’-C), 111.6-112.0 (m, 5’-C, 6-C), 113.8 (4a-C_q_), 119.4 (CN), 127.5 (d, J = 12.9 Hz), 129.7 (5-C), 130.5-130.7 (m, 6’-C), 139.0 (1’-C), 149.2 (8a-C_q_), 159.6 (7-C), 159.8 (2-C), 160.3 (d, J = 247.5 Hz, 4’-C), 162.0 (d, J = 248.8 Hz, 2’-C);* m/z* (%) 314 (43) [M^+^], 201 (100); Anal. calcd. for C_17_H_12_F_2_N_2_O_2_ (%), C, 64.97, H, 3.85, N, 8.91; found: C, 64.90, H, 3.78, N, 8.88.

2-Amino-3-cyano-4-(2’, 3’-difluorophenyl)-7-methoxy-4H-chromene (2M)

Analogously to the synthesis of 2B, compound 2M (100 mg, 0.32 mmol, 32%) was obtained from 2, 3-difluorobenzaldehyde (142 mg, 1.0 mmol), malononitrile (70 mg, 1.0 mmol), three drops of Et_3_N, and 3-methoxyphenol (109 µL, 1.0 mmol) in MeCN (5 mL). Colorless solid of m.p. 183-184 °C.* υ*_max_(ATR)/cm^-1^ 3429, 3331, 3213, 2975, 2935, 2840, 2193, 1652, 1627, 1614, 1576, 1508, 1478, 1416, 1310, 1287, 1266, 1249, 1231, 1190, 1150, 1123, 1043, 1030, 969, 929, 895, 857, 824, 792, 782, 758, 740, 718, 697, 683;^ 1^H NMR (300 MHz, CDCl_3_) 3.76 (3 H, s, OMe), 4.64 (2 H, s, NH_2_), 5.05 (1 H, s, 4-H), 6.52 (1 H, d, J = 2.6 Hz, 8-H), 6.60 (1 H, dd, J = 8.6 Hz, 2.6 Hz, 6-H), 6.9-7.1 (4 H, m, 5-H, 4’-H, 5’-H, 6’-H); ^13^C NMR (75.5 MHz, CDCl_3_) 34.1 (4-C), 55.5 (OMe), 58.9 (3-C), 101.5 (8-C), 111.8 (6-C), 113.5 (4a-C_q_), 116.1 (d, J = 17.2 Hz, 4’-C), 119.3 (CN), 124.3-124.5 (m, 6’-C), 129.7 (5-C), 133.9 (d, J = 9.8 Hz, 5’-C), 148.1 (d, J = 151.0 Hz, (2’-C), 149.2 (8a-C_q_), 151.4 (d, J = 156.0 Hz, 3’-C), 159.7 (7-C), 159.9 (2-C);* m/z* (%) 314 (27) [M^+^], 201 (100); Anal. calcd. for C_17_H_12_F_2_N_2_O_2_ (%), C, 64.97, H, 3.85, N, 8.91; found: C, 64.88, H, 3.80, N, 8.94.

2-Amino-3-cyano-4-(2’, 5’-difluorophenyl)-7-methoxy-4H-chromene (2N)

Analogously to the synthesis of 2B, compound 2N (95 mg, 0.30 mmol, 30%) was obtained from 2, 5-difluorobenzaldehyde (142 mg, 1.0 mmol), malononitrile (70 mg, 1.0 mmol), three drops of Et_3_N, and 3-methoxyphenol (109 µL, 1.0 mmol) in MeCN (5 mL). Colorless solid of m.p. 179-180°C.* υ*_max_(ATR)/cm^-1^ 3629, 3458, 3408, 3333, 3213, 3080, 2983, 2938, 2843, 2192, 1651, 1618, 1579, 1508, 1490, 1452, 1436, 1404, 1317, 1290, 1252, 1240, 1190, 1176, 1150, 1122, 1082, 1044, 1028, 961, 942, 883, 855, 849, 817, 806, 794, 777, 752, 739, 731, 716, 700;^ 1^H NMR (300 MHz, CDCl_3_) 3.76 (3 H, s, OMe), 4.67 (2 H, s, NH_2_), 5.04 (1 H, s, 4-H), 6.52 (1 H, d, J = 2.6 Hz, 8-H), 6.61 (1 H, dd, J = 8.6 Hz, 2.6 Hz, 6-H), 6.8-7.0 (4 H, m, 5-H, 3’-H, 4’-H, 6’-H); ^13^C NMR (75.5 MHz, CDCl_3_) 33.8 (4-C), 55.5 (OMe), 58.8 (3-C), 101.5 (8-C), 111.9 (6-C), 113.4 (4a-C_q_), 115.2-116.2 (m, 4’-C), 116.7-117.2 (m, 3’-C, 6’-C), 119.3 (CN), 129.7 (5-C), 133.1-133.4 (m, 1’-C), 149.2 (8a-C_q_), 156.2 (d, J = 239.8 Hz, 2’-C), 159.0 (d, J = 243.3 Hz, 5’-C), 159.7 (7-C), 159.9 (2-C);* m/ z* (%) 314 (60) [M^+^], 201 (100), 186 (37), 158 (33); Anal. calcd. for C_17_H_12_F_2_N_2_O_2_ (%), C, 64.97, H, 3.85, N, 8.91; found: C, 65.04, H, 3.90, N, 8.98.

2-Amino-3-cyano-4-(3’, 4’, 5’-trifluorophenyl)-7-methoxy-4H-chromene (2P)

Analogously to the synthesis of 2B, compound 2P (105 mg, 0.32 mmol, 32%) was obtained from 3, 4, 5-trifluorobenzaldehyde (160 mg, 1.0 mmol), malononitrile (70 mg, 1.0 mmol) three drops of Et_3_N, and 3-methoxyphenol (109 µL, 1.0 mmol) in MeCN (5 mL). Colorless solid of m.p. 237-238 °C; *υ*_max_(ATR)/cm^-1^ 3428, 3346, 3221, 3053, 3014, 2945, 2848, 2187, 1662, 1616, 1579, 1524, 1506, 1465, 1446, 1409, 1342, 1310, 1292, 1232, 1203, 1182, 1156, 1131, 1116, 1035, 985, 897, 879, 855, 834, 822, 803, 785, 756, 716, 709, 688;^ 1^H NMR (300 MHz, DMSO-d_6_) 3.79 (3 H, s, OMe), 4.81 (1 H, s, 4-H), 6.57 (1 H, d. J = 2.5 Hz, 8-H), 6.69 (1 H, dd, J = 8.7 Hz, 2.5 Hz, 6-H), 6.98 (1 H, d, J = 8.7 Hz, 5-H), 7.07 (2 H, s, NH_2_), 7.1-7.2 (2 H, m, 2’-H, 6’-H); ^13^C NMR (75.5 MHz, CDCl_3_/DMSO-d_6_) 54.8 (3-C), 55.4 (OMe), 101.1 (8-C), 111.5-112.0 (m, 2’-C, 6’-C, 6-C), 113.8 (4a-C_q_), 120.2 (CN), 129.9 (5-C), 136.0 (1’C), 143.3 (4’-C), 148.8 (8a-C_q_), 150.2 (dd, J = 248.7 Hz, 9.8 Hz, 3’-C, 5’-C), 159.2 (7-C), 160.4 (2-C); HR-MS (ESI,* m/z*) for C_17_H_12_O_2_N_2_F_3_ [M^+^ + H] calcd. 333.08454, found 333.08436.

2-Amino-3-cyano-4-(3’-pentafluorothiophenyl)-7-methoxy-4H-chromene (2Q)

Analogously to the synthesis of 2B, compound 2Q (130 mg, 0.32 mmol, 32%) was obtained from 3-pentafluorothiobenzaldehyde (232 mg, 1.0 mmol), malononitrile (70 mg, 1.0 mmol) three drops of Et_3_N, and 3-methoxyphenol (109 µL, 1.0 mmol) in MeCN (5 mL). Colorless solid of m.p. 226-227 °C;* υ*_max_(ATR)/cm^-1^ 3472, 3321, 3281, 3233, 3205, 3071, 2936, 2841, 2192, 1652, 1626, 1612, 1577, 1508, 1464, 1434, 1402, 1293, 1252, 1191, 1155, 1125, 1111, 1097, 1036, 938, 911, 884, 833, 810, 794, 780, 747, 717, 697, 688; ^1^H NMR (300 MHz, CDCl_3_) 3.77 (3 H, s, OMe), 4.64 (2 H, s, NH_2_), 4.75 (1 H, s, 4-H), 6.55 (1 H, d, J = 2.6 Hz, 8-H), 6.62 (1 H, dd, J = 8.6 Hz, 2.6 Hz, 6-H), 6.80 (1 H, d, J = 8.6 Hz, 5-H), 7.3-7.4 (2 H, m, 5’-H, 6’-H), 7.53 (1 H, s, 2’-H), 7.6-7.7 (1 H, m, 4’-H); ^13^C NMR (75.5 MHz, CDCl_3_) 40.5 (4-C), 55.5 (OMe), 60.2 (3-C), 101.6 (8-C), 112.0 (6-C), 113.5 (4a-C_q_), 119.3 (CN), 124.9 (2’-C), 125.3 (4’-C), 129.3 (5’-C), 130.1 (5-C), 131.1 (6’-C), 145.9 (1’-C), 149.2 (8a-C_q_), 154.3-155.0 (m, 3’-C), 159.3 (7-C), 159.8 (2-C);* m/z* (%) 404 (31) [M^+^], 201 (100), 186 (38), 158 (33); Anal. calcd. for C_17_H_12_F_5_N_2_O_2_S (%), C, 50.50, H, 3.24, N, 6.93; found: C, 50.59, H, 3.20, N, 6.88.

2-Amino-3-cyano-4-(3’-bromo-4’, 5’-dimethoxyphenyl)-7-propargyloxy-4H-chromene (3)

A mixture of malononitrile (70 mg, 1.0 mmol) and 3-bromo-4, 5-dimethoxybenzaldehyde (245 mg, 1.0 mmol) in ethanol (EtOH) (5 mL) was treated with three drops of piperidine, and the reaction mixture was stirred for 30 min at room temperature. 3-Propargyloxyphenol (148 mg, 1.0 mmol) was added, and the reaction mixture was stirred under reflux for 4 h. The solvent was evaporated, and the residue was taken up in ethyl acetate, washed with water, and dried over Na_2_SO_4_. The solvent was evaporated, and the residue was purified by column chromatography (silica gel 60). Yield: 50 mg (0.11 mmol, 11%); off-white solid of m.p. 206-207 °C; *R*_f_ = 0.47 (ethyl acetate / *n*-hexane, 1:1);* υ*_max_(ATR)/cm^-1^ 3442, 3331, 3294, 3240, 3202, 3009, 2971, 2939, 2834, 2192, 1651, 1629, 1608, 1570, 1508, 1488, 1459, 1431, 1401, 1312, 1284, 1230, 1183, 1157, 1124, 1044, 1028, 995, 880, 850, 840, 823, 801, 780, 762, 684, 654; ^1^H NMR (300 MHz, DMSO-d_6_) 3.59 (1 H, s, CCH), 3.70 (3 H, s, OMe), 3.81 (3 H, s, OMe), 4.72 (1 H, s, 4-H), 4.80 (2 H, s, CH_2_), 6.65 (1 H, d, J = 2.6 Hz, 8-H), 6.73 (1 H, dd, J = 8.6 Hz, 2.6 Hz, 6-H), 6.88 (1 H, s, 6’-H), 7.0-7.1 (4 H, m, 5-H, 2’-H, NH_2_); ^13^C NMR (75.5 MHz, DMSO-d_6_) 55.4 (CH_2_), 55.7 (3-C), 56.1 (OMe), 60.0 (OMe), 78.6 (C*CH*), 78.9 (*C*CH), 102.5 (8-C), 111.8 (6-C), 112.3 (4a-C_q_), 115.4 (6’-C), 116.8 (3’-C), 120.4 (CN), 122.5 (2’-C), 129.9 (5-C), 143.6 (1’-C), 144.4 (4’-C), 148.6 (5’-C), 153.4 (8a-C_q_), 156.9 (7-C), 160.4 (2-C); HR-MS (ESI,* m/z*) for C_21_H_18_O_4_N_2_^79^Br [M^+^ + H] calcd. 441.04445, found 441.04419.

### Biological assays

#### Cell lines and culture conditions

The following cell lines were used: 518A2 melanoma (Department of Radiotherapy, Medical University of Vienna, Austria)^[15]^, HeLa cervix carcinoma, KBV1^Vbl^ (ACC-149 cervix carcinoma, MCF7 [ACC-115]) breast carcinoma, U-87 glioblastoma, HT-29 (ACC-299), HCT-116 (ACC-581) and HCT-116p53-/- colon carcinoma, EA.hy926 (ATCC® CRL-2922^TM^) endothelial hybrid cells, and HDFa (ATCC® PCS-201-012^TM^) adult human dermal fibroblasts. The cells were maintained in Dulbecco’s Modified Eagle Medium with 10% fetal bovine serum (20% for HDFa cells) and 1% ZellShield® at 37 °C, 5% CO_2_, and 95% humidity. KB-V1^Vbl^ cells were treated with 340 nM vinblastine to retain vinblastine resistance. Only mycoplasma-free cultures were used.

#### In vitro cytotoxicity assay

An 3-(4, 5-dimethylthiazol-2-yl)-2, 5-diphenyl tetrazolium bromid (MTT) assay was used for antiproliferative studies. Cancer cells (5 × 10^4^ cells/mL, 100 μL/well) were placed in 96-well plates (10 × 10^4^ cells/mL for HDFA and U87 cells), and the cells were incubated for 24 h at 5% CO_2_ and 95% humidity. Then, cells were treated with test compounds (at various concentrations) or solely with DMSO as a negative control for 72 h. Then, 12.5 μL of a 0.5% MTT solution in phosphate-buffered saline (PBS) was added to the cells, followed by incubation for 2 h at 37 °C. After centrifugation of the plates (300 × g, 5 min, 4 °C), the medium was discarded, and 25 μL of DMSO containing 10% SDS and 0.6% acetic acid was added, followed by incubation at 37 °C for at least 1 h. Formazan absorbance at λ = 570 nm was measured using a Tecan infinite F200 microplate reader and corrected for the background (λ = 630 nm). The 50% inhibitory concentration (IC_50_) values were calculated from dose-response curves (means ± SD, four independent experiments) compared to DMSO-treated control cells, which were set to 100%. GraphPad Prism 9 was used for curve-fitting.

#### Microtubule immunofluorescence staining

518A2 melanoma cells (10 × 10^4^ cells/mL, 0.5 mL/well) were placed on coverslips in 24-well plates followed by incubation at 37 °C, 5% CO_2_, and 95% humidity. The cells were treated with test compounds and controls (25 and 100 nm) for 0.5, 1, 3, and 6 h and washed with cytoskeletal buffer (100 nm PIPES, 3 nm MgCl_2_, 138 nm KCl, 2 nm EGTA, 300 nm sucrose, pH 6.8). Fixation and permeabilization were performed for 5 min with 3.7% formaldehyde and 0.2% Triton X-100 in a cytoskeletal buffer. The cells were fixed with cold EtOH for 10 s, rehydrated in PBS, and blocked with 1% bovine serum albumin in PBS for 30 min. The microtubules were stained for 1 h with primary (anti-α-tubulin, mouse monoclonal antibody) and secondary antibodies (goat anti-mouse IgG-AF-546, Invitrogen) and washed with PBS between each treatment. Nuclei were stained using DAPI (1 µg/mL in PBS) for 30 min. The coverslips were placed in Roti®-Mount FluorCare. Microtubules (λ_ex_ = 488 nm, λ_em_ = 507 nm) and nuclei (λ_ex_ = 358 nm, λ_em_ = 461 nm) were documented by confocal microscopy (Leica TCS SP5 confocal microscope, 630 × magnification) and edited with *ImageJ.*

#### Intracellular localization

518A2 melanoma cells (7.5 × 10^4^ cells/mL, 0.5 mL/well) were placed on coverslips in 24-well plates and cultivated for 24 h. Cells were treated with **3** (25 µm, 0.4% tween 80 in PBS) for 10 min at room temperature. After fixation in 3.7 % formaldehyde for 15 min and washing with PBS, the cells were incubated with the “click-solution” (2 mm CuSO_4_, 5 mm sodium ascorbate, 0.1 mm 3-azido-7-hydroxycoumarin, 1% bovine serum albumin in PBS) for 30 min and washed with PBS. The nuclei were counterstained with Nuclear Green LCS1 for another 30 min, and the coverslips were mounted in Roti®-Mount FluorCare. The clicked test compound (λ_ex_ = 404 nm, λ_em_ = 477 nm) and nuclei (λ_ex_ = 488 nm, λ_em_ = 507 nm) were analyzed with a Leica TCS SP5 confocal microscope, and the obtained images were edited with *ImageJ*.

#### Cell-cycle analysis

518A2 melanoma cells (10 × 10^4^ cells/mL, 3 mL/well) were cultivated in six-well plates for 24 h. After the treatment with test compounds (10 and 25 nm), C-A4 (10 and 25 nm), or DMSO (vehicle) for an additional 24 h, the cells were trypsinized, centrifuged (300 × *g*, 5 min, 4 °C), and fixed in 70% EtOH for min. 24 h. For FACS measurement, cells were washed in PBS and stained with propidium iodide solution (50 µg/mL PI, 50 µg/mL RNase A in 0.1% sodium citrate) for 30 min at 37 °C. The DNA content of at least 10000 single cells was determined using a Beckmann Coulter Cytomics FC500 flow cytometer (λ_ex_ = 488 nm, λ_em_ = 570 nm). CXP software (Beckman Coulter) was used to analyze the cells in the cell cycle phases (sub-G1, G1, S, G2/M).

#### Tube-formation assay

EA.hy926 endothelial hybrid cells were maintained for 24 h in EndoPrime low-serum endothelial medium and seeded (3 × 10^5^ cells/mL, 50 µL/well) on basement membrane-like matrix Matrigel® on Ibidi µ-Slides. After treatment with test compounds for 4 h until tubular structures had formed in the control wells, results were documented using a Zeiss Axiovert 135 light microscope. Cell vitality was measured using the MTT assay and was above 75% compared to solvent-treated cells. Experiments were performed in triplicate.

#### Zebrafish angiogenesis assay

Transgenic zebrafish (*Tg(fli1:EGFP, casper *mutant) were bred at 28 °C.^[16]^ After fertilization, the eggs were transferred to E3-medium (5 mm NaCl, 0.17 mm KCl, 0.33 mm CaCl, 0.33 mm MgSO_4_, 0.01% methylene blue, pH 7.2), followed by incubation for 24 h. The chorion was manually removed, and the larvae were treated with test compounds or solvent in six-well plates (30 fish per concentration, 5 mL/well) for 48 h. The fluorescent vasculature was documented with a Leica MZ10F and Zeiss AxioCam MRrc. To quantify the angiogenesis, the sub-intestinal vein (SIV) area was measured using ImageJ and expressed as means ± SD with the control set to 100%. The significance of SIV reduction through substance treatment was assessed using one-way analysis of variance (ANOVA), *P < 0.05; **P < 0.01; ***P < 0.001; ****P < 0.0001, with Dunnett´s multiple comparison test (GraphPad Prism 9).

#### Luciferase-dependent MYB activity assay

Compounds were tested using the HEK-MYB-Luc reporter cell line, which allows a doxycycline-dependent induction of MYB expression and harbors an MYB-dependent luciferase reporter plasmid, as previously described^[17,18]^. After 16 h of substance treatment (0.0001-3 µM), luciferase activities were analyzed as described^[11]^.

## RESULTS

**Scheme 1:**
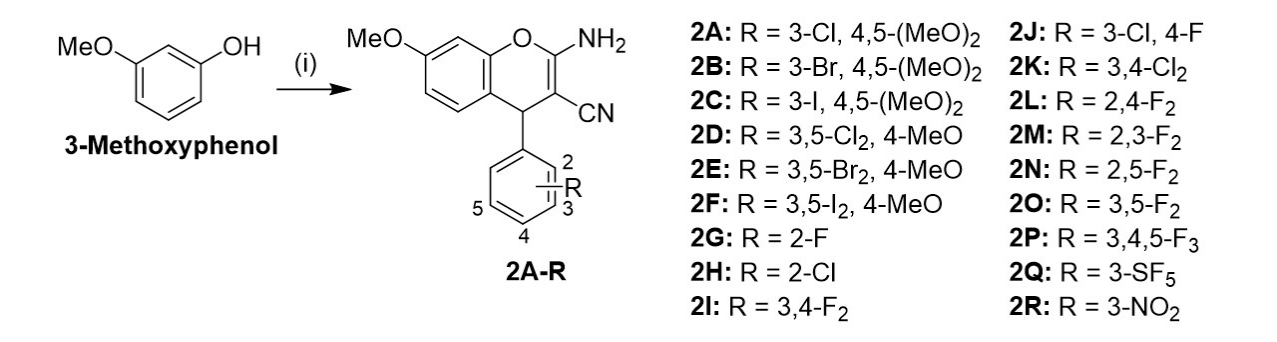
Reagents and conditions: (i) Malononitrile; aryl aldehyde; cat. Et_3_N; MeCN; rt; 3-16 h.

The test compounds 2A-R were obtained from 3-methoxyphenol, malononitrile, the corresponding aryl aldehyde, and a cat. amount of Et_3_N in acetonitrile [Scheme 1]. Synthesis and analysis of the known compounds 2B, 2O, and 2R were as described^[5,14]^. The new compounds 2A, 2C-N, 2P, and 2Q were colorless solids. The structures of the test compounds were confirmed by NMR, IR, and MS analyses. The compounds are racemic mixtures, and no efforts to separate enantiomers were performed. The yields were generally low but acceptable considering the simple one-pot reaction and workup and the commercially available starting compounds [Table 1].

**Table 1:** Yields of the syntheses of compounds 2A-R

**Compound**	**Yield**	**Compound**	**Yield**	**Compound**	**Yield**
2A	22%	2G	25%	2M	32%
2B	30%	2H	40%	2N	30%
2C	32%	2I	31%	2O	20%
2D	31%	2J	30%	2P	32%
2E	31%	2K	30%	2Q	32%
2F	30%	2L	32%	2R	30%

**Scheme 2:**
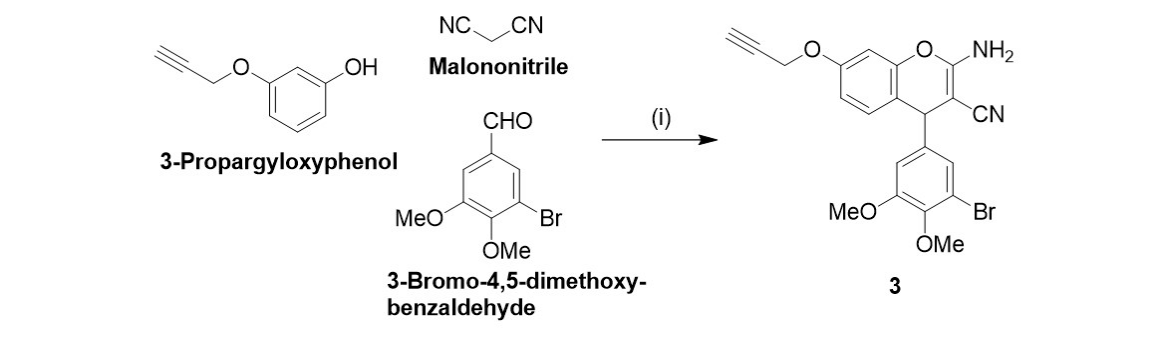
Reagents and conditions: (i) cat. piperidine; EtOH; reflux; 5 h; 11%.

The new propargyl ether derivative 3 was prepared to conduct localization studies of this compound within cancer cells [Scheme 2]. 3-Propargyloxyphenol was obtained from the reaction of resorcinol with 1.2 equiv. propargyl bromide (80% in toluene) in DMF under basic conditions (K_2_CO_3_)^[19]^. Harsher conditions (reflux for 5 h in EtOH) were necessary to synthesize 3 by the described three-component reaction compared with the synthesis conditions of compounds 2A-R. 3 was obtained as an off-white solid in low yields only.

The antiproliferative activity of compounds 2A-R and 3 was evaluated in nine tumor and hybrid cell lines from six entities and compared with previously published data of the known compounds 1A and 1B [Table 2]^[13,20]^.

**Table 2:** IC_50_ values (in nM) of 2A-R and 3 in tumor cell lines.^[a]^1A (LY290181) and 1B (Bcr-TMP) were used as positive controls

	**EA.hy926**	**518A2**	**HCT-116**	**HCT-116 p53-/-**	**U87**	**HT-29**	**KB-V1^Vbl^**	**KB-V1^Vbl^^[b]^**	**HeLa**	**MCF-7**	**HDFa**
1A^[c]^	30 ± 1	10 ± 1	8 ± 0.7	30 ± 3	70 ± 9	30 ± 3	20 ± 2	80 ± 2	40 ± 2	200 ± 20	40,700 ± 1500
1B^[c]^	20 ± 1	30 ± 0.1	20 ± 1.9	30 ± 2	5 ± 0.2	300 ± 40	30 ± 1	9 ± 1	10 ± 1	34 ± 2.5	-
1C^[d]^	2800 ± 70	1500 ± 100	3100 ± 100	2900 ± 60	5500 ± 300	2500 ± 200	3700 ± 60	3500 ± 80	-	3400 ± 200	-
2A	0.4 ± 0.06	4 ± 0.3	70 ± 5	70 ± 5	20 ± 3	300 ± 20	6 ± 0.5	0.9 ± 0.08	10 ± 0.4	40 ± 7	11,500 ± 1200
2B	0.8 ± 0.09	6 ± 0.2	100 ± 10	30 ± 3	80 ± 5	200 ± 20	10 ± 1	0.7 ± 0.06	2 ± 0.2	100 ± 8	> 50,000
2C	60 ± 5	20 ± 2	70 ± 1	6 ± 0.7	25 ± 2	200 ± 20	60 ± 5	3 ± 0.3	10 ± 0.9	20 ± 1	> 50,000
2D	1100 ± 70	2100 ± 200	2500 ± 200	1900 ± 200	3900 ± 400	1400 ± 100	2100 ± 100	2400 ± 100	700 ± 50	2400 ± 300	-
2E	20 ± 1	20 ± 2	20 ± 2	40 ± 3	20 ± 2	300 ± 2	3200 ± 300	10 ± 0.5	30 ± 3	80 ± 3	22,200 ± 2100
2F	20 ± 2	20 ± 0.4	30 ± 5	30 ± 1	50 ± 10	60 ± 1	70 ± 7	40 ± 2	30 ± 3	80 ± 4	> 50,000
2G	900 ± 90	700 ± 40	200 ± 20	300 ± 40	3200 ± 100	200 ± 10	700 ± 20	500 ± 20	400 ± 60	500 ± 40	-
2H	3700 ± 300	2000 ± 100	3700 ± 400	2800 ± 300	7400 ± 200	5300 ± 970	1900 ± 200	1600 ± 200	1400 ± 100	5100 ± 370	-
2I	700 ± 10	700 ± 5	800 ± 6	600 ± 6	3600 ± 500	800 ± 30	700 ± 30	60 ± 5	600 ± 30	700 ± 100	-
2J	400 ± 20	400 ± 10	300 ± 30	400 ± 40	1200 ± 70	500 ± 50	300 ± 20	210 ± 10	100 ± 6	150 ± 20	-
2K	600 ± 80	600 ± 7	300 ± 10	800 ± 20	8500 ± 300	1900 ± 100	1300 ± 100	600 ± 50	800 ± 70	1000 ± 50	-
2L	200 ± 10	500 ± 30	2500 ± 40	1600 ± 160	5100 ± 500	500 ± 50	3800 ± 400	1000 ± 100	1500 ± 70	> 50,000	-
2M	400 ± 10	300 ± 10	500 ± 50	200 ± 8	400 ± 60	200 ± 20	900 ± 90	100 ± 4	90 ± 2	200 ± 30	-
2N	4700 ± 900	90 ± 60	500 ± 50	200 ± 10	500 ± 100	400 ± 30	700 ± 70	200 ± 20	200 ± 20	300 ± 7	-
2O	40 ± 1	60 ± 6	1400 ± 50	40 ± 3	100 ± 10	70 ± 8	20 ± 2	60 ± 4	40 ± 4	400 ± 60	-
2P	400 ± 40	300 ± 30	300 ± 20	300 ± 20	1100 ± 90	200 ± 5	100 ± 5	100 ± 10	100 ± 20	400 ± 50	-
2Q	600 ± 20	300 ± 10	700 ± 40	2900 ± 10	900 ± 60	400 ± 30	1900 ± 100	2000 ± 100	400 ± 4	800 ± 30	-
2R	300 ± 9	30 ± 2	300 ± 30	200 ± 20	900 ± 50	200 ± 20	30 ± 0.4	70 ± 6	300 ± 20	10 ± 0.8	-
3	80 ± 2	60 ± 3	300 ± 10	90 ± 7	90 ± 3	2100 ± 300	80 ± 3	100 ± 3	40 ± 2	70 ± 4	-

^[a]^Means of min. four independent experiments (± SD); ^[b]^plus verapamil; ^[c]^Köhler *et al.*^[20]^; ^[d]^Köhler *et al.*^[13]^.

The presentation of the cytotoxicity screening results was simplified by determining the average IC_50_ values of all cell lines and sorting them based on their antiproliferative activity [Table 3]. The halogen atom did not have any major effect on the activity among the compounds with a 3-halo-4, 5-dimethoxyphenyl group (2A, 2B, and 2C). By contrast, in the group of 3, 5-dichloro- to 3, 5-diiodo-4-methoxyphenyl derivatives 2D, 2E, and 2F, a distinct increase in activity was observed from the dichloro compound 2D via the dibromo 2E to the diiodo derivative 2F. Several striking effects were observed in addition to this trend. Comparing the structure of 1C (coumarin-based) and 2F (7-methoxy-4*H*-chromene), a clear increase in antiproliferative activity based on the methoxychromene structure was observed, while the IC_50_ values of the 4*H*-naphtho(1, 2-*b*)pyran-3-carbonitriles (1A) and the 7-methoxy-4*H*-chromenes (2R) were both in the nanomolar range. Some compounds showed certain tumor cell line-specific activities, surpassing positive controls 1A and 1B. 2C was particularly active against HCT116 p53-deficient colon carcinoma cells, verapamil-treated KB-V1^Vbl^ cervix carcinoma cells and MCF-7 breast cancer cells, whereas 2F and 2O were highly active against multidrug-resistant HT-29 colon carcinoma cells. The 1A-analog 2R showed slightly less overall activity but had a much stronger effect on MCF-7 breast cancer cells than 1A. The vinblastine-resistant KB-V1^Vbl^ cells were treated with the P-glycoprotein (P-gp) blocker verapamil to identify synergy effects^[21]^. Compounds 2I, 2B, 2C, and 2E showed 12-, 14-, 20- and 320-fold lower IC_50_ values combined with verapamil (1 µM), assuming an inhibition of the efflux pumps increases the substance efficiency by blocked drug removal via the P-gp membrane transporter. A comparison of compounds 2B and 3, prepared for intracellular localization purposes, showed a substantial conformity of activity, indicating a similar mode of action and accumulation behavior in most tumor cell lines. However, compound 2B was more active against verapamil-treated KB-V1^Vbl^ cells than against untreated cells and HeLa cells (which is the parent cell line of KB-3-1, from which KB-V1^Vbl^ cells are derived), while 3 was more active against HeLa cells and KB-V1^Vbl^ cells in the absence of verapamil than against verapamil-treated KB-V1^Vbl^ cells.

**Table 3:** Average IC_50_ value [nM] of the tested cancer and hybrid cell lines for compounds 1A-B, 2A-R, and 3, and SI (IC_50_ HDFa cells/IC_50_ tumor cell average)

	**2F**	**2C**	**2B**	**1A**	**2A**	**1B**	**2O**	**2R**	**3**	**2M**	**2P**	**2E**	**2J**	**2G, 2N, 2J, 2Q, 2K, 2D, 1C, 2H, 2L**
Ø IC_50_ [nm]	43	47	49	52	53	53	203	227	321	330	330	374	396	> 500
SI	1163	1055	952	786	219	-	-	-	-	-	-	59	-	-

The most active derivatives 2A-C and 2E-F and the positive control 1A were tested for their toxic effects on non-malignant HDFa cells [Table 3]. The selectivity index (SI) was calculated as a measure of selectivity toward cancer cells compared to non-malignant cells. 2B, 2C, and 2F displayed exceptionally high SI values, highlighting their potential as anticancer drug candidates.

**Figure 2:**
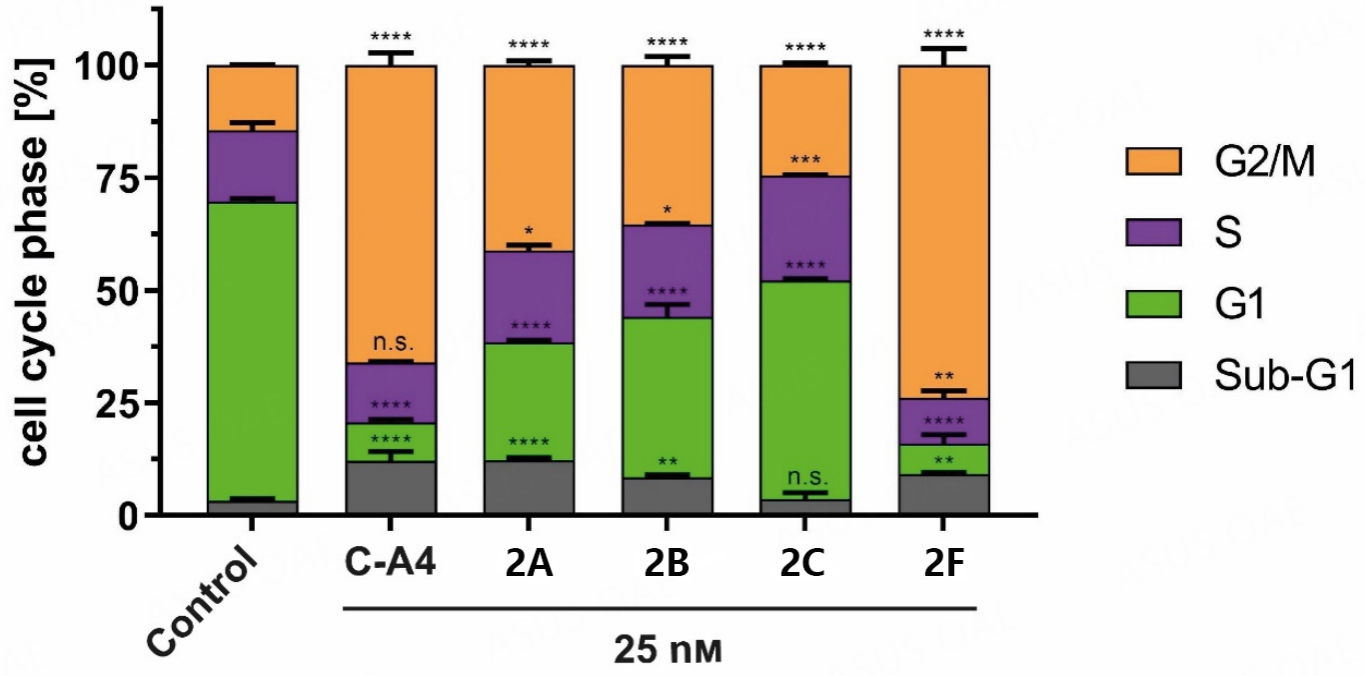
Cell cycle events of 518A2 melanoma cells treated with 2A-C and 2F (25 nM) for 24 h. Positive control (C-A4) and solvent (DMSO) were treated similarly to the substances. Measurements were carried out in triplicate and expressed as means ± SD with GraphPad Prism. Significance is expressed as n.s. *P* > 0.05; *P < 0.05; **P < 0.01; ***P < 0.001; ****P < 0.0001 against control (two-way ANOVA, with Dunnett’s multiple comparison test).

For a more detailed investigation of possible drug mechanisms, compounds 2A-C and 2F were tested for their influence on the cell cycle of 518A2 melanoma cells. FACS analysis at doses of 25 nM revealed a significant G2/M cell-cycle arrest for these compounds and the positive control combretastatin A4 (CA4), but to varying extents [Figure 2].

Among the compounds 2A-C, the 3-chloro-4, 5-dimethoxyphenyl derivative 2A showed more significant cell-cycle arrest than its 3-bromo and 3-iodo congeners 2B and 2C. However, an opposite effect was observed for the 3, 5-dihalo-4-methoxyphenyl derivatives 2D-F, where the 3, 5-diiodo-4-methoxyphenyl derivative 2F triggered by far the most significant arrest. The analogous 3, 5-dichloro (2D) and 3, 5-dibromo (2E) compounds induced only a slight increase in G2/M phase cells (data not shown). Substance 2F arrested about 74% of cells before or during mitosis and exceeded the C-A4 control. C-A4, 1A, and 1B caused G2/M arrest by microtubule depolymerization, thus preventing the formation of a functional spindle apparatus required for cell division^[7,11,22]^.

**Figure 3:**
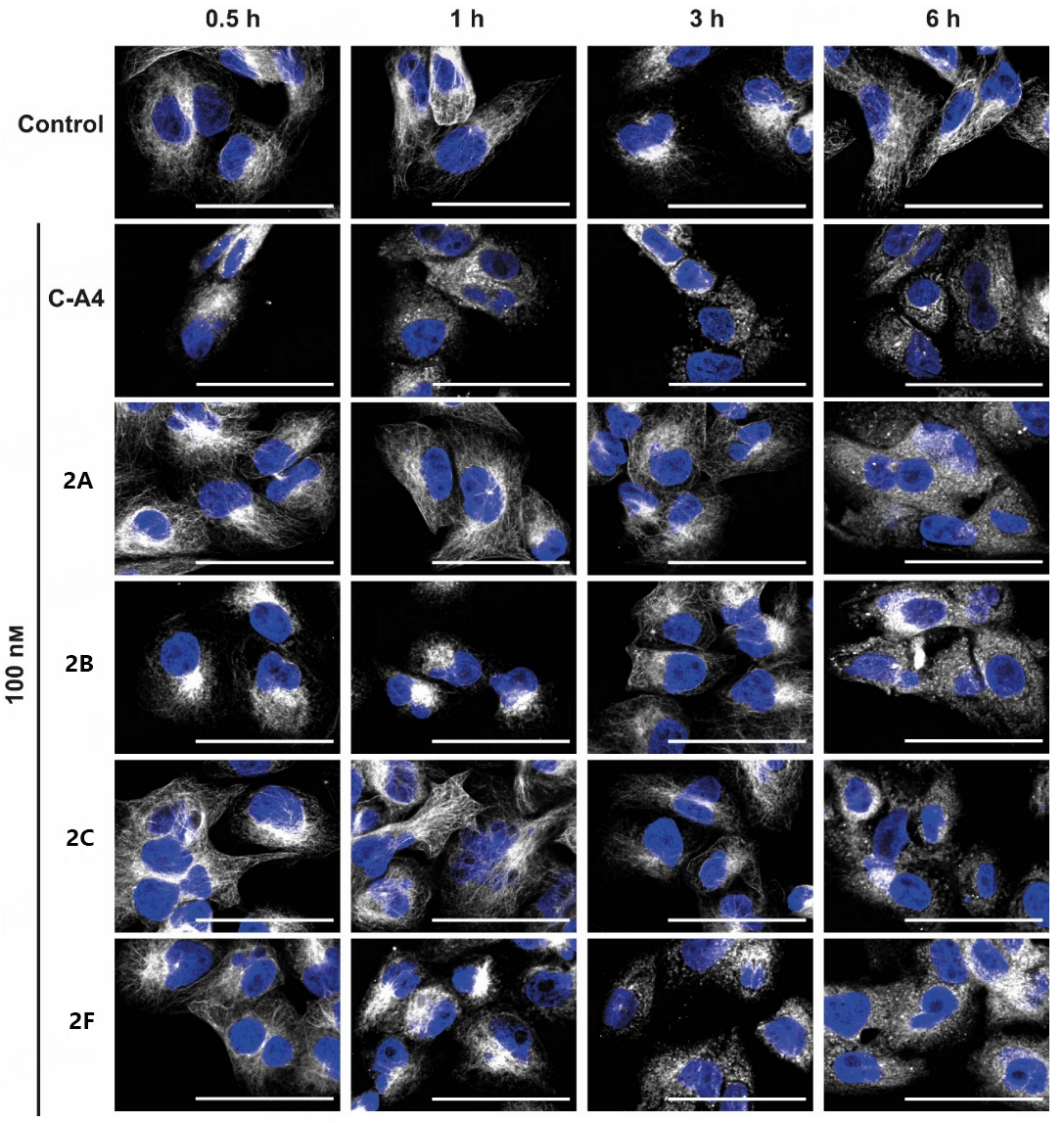
Immunofluorescence images of 518A2 melanoma cells treated with compounds 2A-C; 2F; and CA4 (100 nM) or vehicle (DMSO) for 0.5, 1, 3, and 6 h. Representative images (of two experiments) illustrate stained microtubules (white) and nuclei (blue). The scale bar corresponds to 100 µm, magnification of 630×.

Because of the structural similarity of the new cell cycle arresting compounds 2A-C and 2F to the known tubulin-depolymerizing agent C-A4, the effects on the microtubule cytoskeleton were investigated. Time-dependent imaging of 518A2 melanoma cells was applied to provide insights into the dynamics of the depolymerization process [Figure 3].

In solvent-treated cells, the tubulin cytoskeleton consists of fine filaments extending throughout the cytoplasm. Upon treatment with test compounds 2A-C and 2F, these filaments were initially shortened and fragmented, leading to their destruction and distribution within the cytoplasm. However, the timing of this disintegration process was highly dependent on the substance used. For instance, 2A-C showed a breakdown of the cytoskeleton after 6 h, whereas 2F led to a change in the microtubule structure after 1 h. A similar effect was observed for the C-A4 control after 30 min, which can be attributed to a faster uptake or higher target affinity.

**Figure 4:**
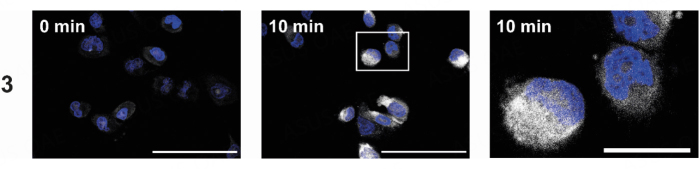
Intracellular localization of 3 (25 µM) using melanoma cells (518A2) after 10 min. The uptake was visualized using a Cu(I)-catalyzed reaction with 3-azido-7-hydroxycoumarin (white), and the nuclei were counterstained (Nuclear Green, blue). The right image shows the magnified section marked with a white box. The experiment was carried out in duplicate. The scale bar corresponds to 100 µm (left) or 25 µm (right), magnification of 630×.

The alkynyl derivative 3 was designed to investigate compound uptake and localization within tumor cells. The propargyl group allows orthogonal fluorescence labeling with 3-azido-7-hydroxycoumarin using a copper-catalyzed click reaction. After 10 min, a distinct increase in fluorescence was observed within the treated cells, with most of the fluorescence found in the cytoplasm [Figure 4]. This finding suggests an accumulation of 3 in the cytoplasm, supporting the hypothesis that cytoplasmic tubulin is the primary target for 3 and its close analogs used in this study. The conformity of the basic structure and the antiproliferative activity of 3 with compound 2 suggests a similar mechanism.

**Figure 5:**
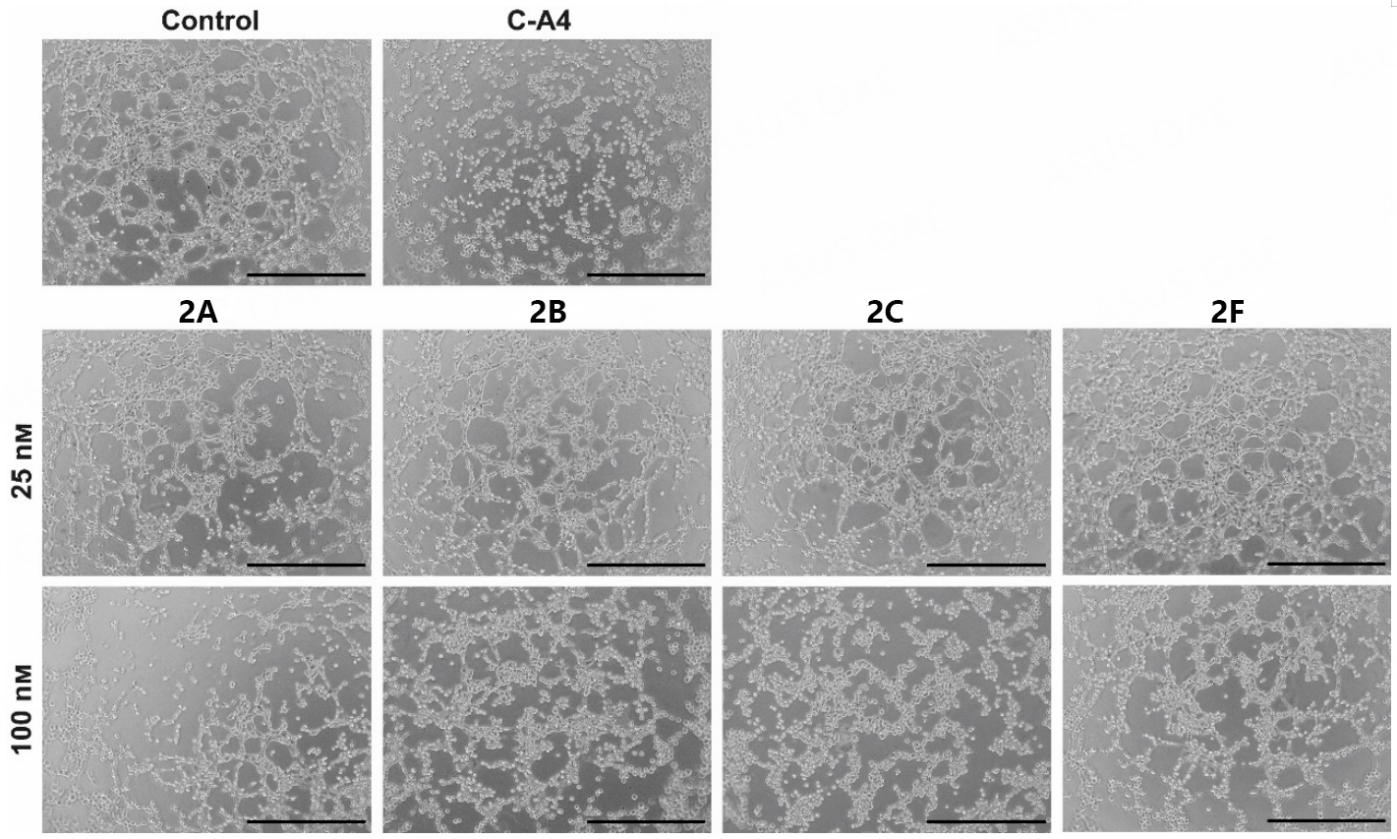
Images show EA.hy926 cells seeded on Matrigel® after 4 h treatment with substances 2A-C; 2F (25, 100 nM); and C-A4 (25 nM) or vehicle (DMSO). Representative images of a min. of two experiments. The scale bar corresponds to 500 µm, magnification of 100×.

In addition to antiproliferative and cytotoxic effects, microtubule-destabilizing agents possess additional antitumor properties^[23]^. In the case of C-A4, anti-angiogenic and vascular disruptive effects have been demonstrated. In this context, the tube-formation assay is a suitable method to observe the effects on the two-dimensional (2D) vessel structures by EA.hy926 cells^[24]^. The inhibition of cell migration and the development of cell-cell junctions (necessary for tube formation) by 2A-C and 2F were investigated [Figure 5]. At 100 nM, all four test compounds showed anti-angiogenic effects on EA.hy926 cells seeded on Matrigel®. The cells could not form a cross-linked 2D structure within 4 h, as with the negative control or at 25 nM substance concentration. Even if cell-cell junctions were formed in isolated cases, most cells agglomerated due to their spatial proximity. In addition, rounding was observed in many cells, as in C-A4 treated cells, presumably because of microtubule-destabilizing effects.

**Figure 6:**
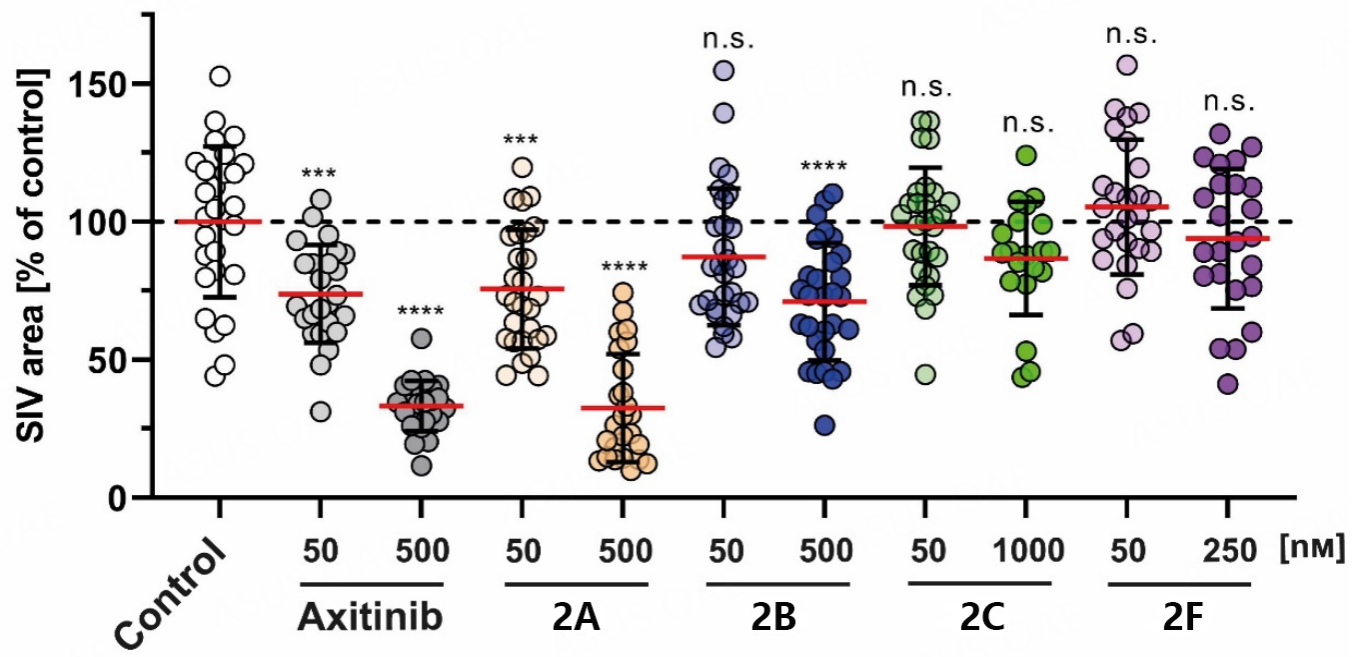
Effects of 2A; 2B (50, 500 nM); 2C (50, 1000 nM); and 2F (50, 250 nM) on the SIV growth of zebrafish larvae (24 hpf) after treatment (48 h). Positive controls used axitinib (50, 500 nM). Negative controls used equivalent amounts of DMSO. The SIV area was quantified using ImageJ and expressed as mean ± SD of at least 20 zebrafish. The significance is expressed as n.s. *P* > 0.05; ***P < 0.001; ****P < 0.0001 against control (one-way ANOVA, with Dunnett´s multiple comparison test).

The development of blood vessels is based on a complex mechanism with various regulators, which are essential targets for the treatment of tumor growth. Angiogenesis can be seen in the embryonal development of zebrafish larvae, where the SIV can be used to measure anti-angiogenic effects^[25]^. After exposure of 24-h-old zebrafish embryos to substances 2A-C and 2F or positive control axitinib for 48 h, we measured the SIV area and compared it to solvent-treated fish [Figure 6]. Here, 2F showed no significant change in blood vessel growth, and 2F had a toxic effect above 250 nM. 2B and 2C showed no SIV decrease in the tolerated concentration range. However, 2A inhibited angiogenesis to an extent comparable to the known inhibitor axitinib^[26]^. This finding suggests a different mechanism of action for 2A, a bimodal compound with selective antiproliferative properties against cancer cells and angiogenesis-inhibiting features.

**Figure 7:**
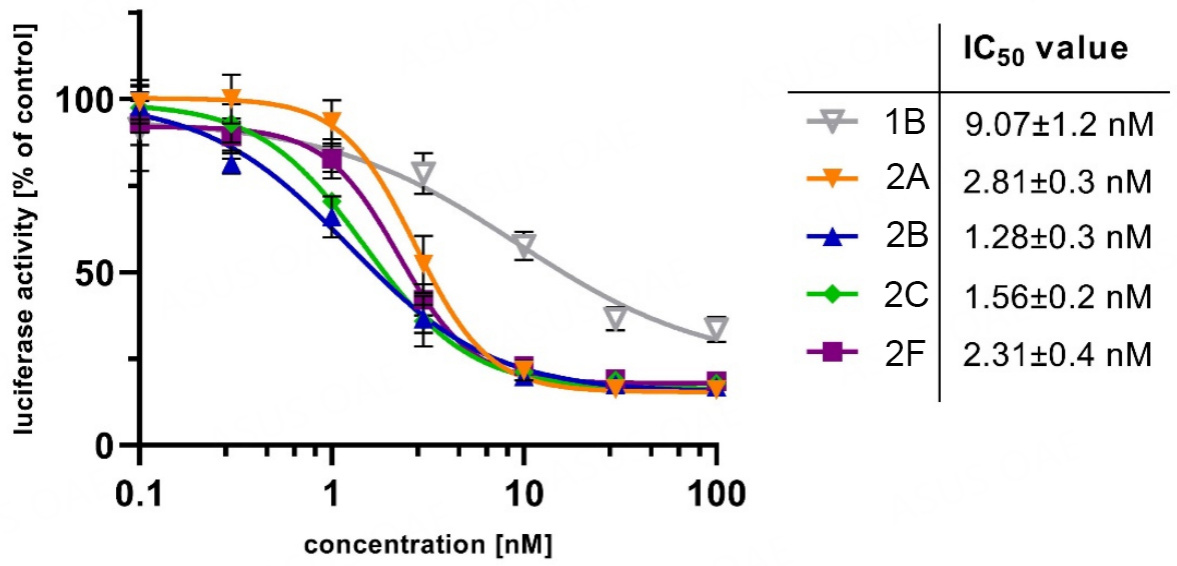
Inhibition of MYB activity in HEK-293 cells containing the reporter plasmid pGL4-5xMRE(GG)-Myc and the expression vector for MYB-2KR, upon treatment with compounds 1B; 2A-C; and 2F (0.1-100 nM) for 16 h. IC_50_ values were calculated with at least four independent experiments using GraphPad Prism 9.

Due to their structural similarity to the potent MYB inhibitor 1B (data to be published elsewhere), compounds 2A-C and 2F were tested for their MYB-inhibitory activity [Figure 7]. Inhibition of MYB activity by 2A-C and 2F (1.28-2.81 nM) was superior to the inhibition by reference compound 1B (9.07 nM). Because the values are very close, it is challenging to identify a structure-dependent activity trend; however, the activity was in the order 2B > 2C > 2F > 2A.

## DISCUSSION

This study’s objective was to develop and optimize the lead structures 1A, 1B, and 1C. For this reason, we reduced the size of the benzo[*h*]chromene backbone to a 7-methoxy-4*H*-chromene structure, which led to increased activity. In addition, various phenyl substituents were tested to optimize the lead structure. Initial cytotoxicity studies against nine cancer cell lines confirmed that the C-A4-derived 3-iodo-, 3-bromo-, and 3-chloro-4, 5-dimethoxyphenyl motifs (2A-C) and the analogous 3, 5-diiodo- and 3, 5-dibromo-4-methoxyphenyl derivatives (2E-F) are excellent pharmacophores with selectivity against malignant cells. Only the 3, 5-dichloro-4-methoxyphenyl stands out with only micromolar IC_50_ values. Interestingly, differences in the specificity for HeLa cells, derived KB-V1^Vbl^ cells, and KB-V1^Vbl ^cells treated with the P-gp inhibitor verapamil were observed for the close analogs 2B and 3. 2B is more active against the parent HeLa cells than against the vinblastine-resistant KB-V1^Vbl^ cells. In contrast, the addition of verapamil strongly sensitized the KB-V1^Vbl^ cells to treatment with 2B, leading to a higher activity of 2B against verapamil-treated KB-V1^Vbl^ cells than against HeLa cells. A comparable hypersensitizing effect of verapamil was observed for KB-V1^Vbl^ cells treated with 2A, 2C, and 2I, surpassing their activity against HeLa cells, which may have explanations beyond mere P-gp inhibition by verapamil. The hypersensitivity effects of resistant P-gp-overexpressing cancer cells upon verapamil treatment were reported (e.g., based on disrupted energy homeostasis upon ATP depletion), which might lead to enhanced anticancer activity combined with other active drug candidates such as 2A-C (but not 2F) compared with their activity against related cell lines without (overexpressed) P-gp transporter^[27]^. For unknown reasons, compound 3 showed only slightly reduced activity against KB-V1^Vbl^ cells when combined with verapamil than against HeLa cells.

In addition, a reversal of P-gp- and BCRP-mediated resistance was documented for the 3-chloro-4-fluoroanilino-derivative gefitinib, an approved EGFR inhibitor^[28,29]^. However, the new 3-chloro-4-fluorophenyl derivative 2J appears to be a substrate of P-gp. Among the remaining fluorophenyl derivatives, 2, 3-difluorophenyl 2M and 2, 5-difluorophenyl 2N were identified as P-gp substrates, while 3, 5-difluorophenyl 2O and 3, 4, 5-trifluorophenyl 2P showed no dependence on P-gp, indicating a considerable influence of the fluoro-substitution pattern on the activity against P-gp-overexpressing cells (also compared with the effects of 3, 4-difluorophenyl derivative 2I mentioned above).

Because P-gp tends to have relatively hydrophobic substrates with aromatic rings, substituting methoxy groups with halogen atoms may already have an efflux-attenuating effect due to increased hydrophilicity^[30]^. Treatment with P-gp modulators can provide more significant cytotoxicity by reducing effective concentrations by 100-fold (paclitaxel) or 351-fold (vinblastine) in MDR colorectal cancer SW620/Ad300 cells^[31,32]^.

The test compounds were obtained and tested as racemic mixtures. An increase in activity might be achieved by the separation of the enantiomers of the active derivatives and evaluating the separated enantiomers via MTT assay to identify any enantiomers which are more active or less active than the mixture. Instead of separating racemic mixtures, chiral synthetic procedures (e.g., using chiral organic bases instead of piperidine or triethylamine) might be applied to generate enantiopure compounds for biological testing.

The evaluation of the four most active test compounds (2A-C and 2F) revealed a correlation between the rate of microtubule destruction and the cell-cycle arrest in the G2/M phase in 518A2 melanoma cells. Compared with C-A4, 2F showed almost equal efficacy and led to early morphological changes in the microtubules (i.e., within 1 h). Localization of the structurally related alkynyl derivative 3 in the cytosol confirmed the accumulation near the cytoplasmic target. As previously demonstrated for the controls 1C and C-A4, the influence on angiogenesis (essential for tumor growth and metastases) was investigated for the test substances^[13,33]^. Using the 2D tube-formation assay, the concentration-dependent impairment of cell motility and intercellular junctions could be demonstrated, probably due to the damaged tubulin cytoskeleton. Nevertheless, this model can only partially simulate the complex mechanisms of blood vessel formation, which is why the substances were also tested for anti-angiogenic effects in zebrafish. This assay offers the possibility of estimating toxicity in a vertebrate and investigating the direct influence on the angiogenesis of the so-called SIVs. Surprisingly, only 2A showed a significant decrease in SIV growth, which occurred independently of its antiproliferative and microtubule-associated effects. As a third potential target, the inhibitory effect on the transcription factor MYB was tested and revealed the enhancement by three- to seven-fold compared to the previously discovered inhibitor 1B^[11]^. Overexpression of MYB in leukemias and various solid cancers such as colon and ER-positive breast cancers contributes to their development and thus represents a valuable target^[34-39]^. The rapid development of resistance to selective chemotherapeutics (e.g., kinase inhibitors, including RAF inhibitors) is a problem that can be overcome by addressing various targets by drugs with dual or multimodal mechanisms of action such as dual BRAF/HDAC inhibitors^[40,41]^. In this context, compound 2A represents a promising scaffold that can be used for further optimization and advanced stages of preclinical anticancer testing.

## DECLARATIONS

### Availability of data and materials

Data supporting the findings of this study are available from the authors upon request.

### Copyright

© The Author(s) 2023.

## References

[1] Fouad MA, Abdel-Hamid H, Ayoup MS (2020). Two decades of recent advances of Ugi reactions: synthetic and pharmaceutical applications. RSC Adv.

[2] Nagarajaiah H, Mukhopadhyay A, Moorthy JN (2016). Biginelli reaction: an overview. Tetrahedron Lett.

[3] Zheng X, Liu W, Zhang D (2020). Recent advances in the synthesis of oxazole-based molecules via van leusen oxazole synthesis. Molecules.

[4] Wang L, Woods KW, Li Q (2002). Potent, orally active heterocycle-based combretastatin A-4 analogues: synthesis, structure-activity relationship, pharmacokinetics, and in vivo antitumor activity evaluation. J Med Chem.

[5] Kemnitzer W, Kasibhatla S, Jiang S (2005). Discovery of 4-aryl-4H-chromenes as a new series of apoptosis inducers using a cell- and caspase-based high-throughput screening assay. 2. Structure-activity relationships of the 7- and 5-, 6-, 8-positions. Bioorg Med Chem Lett.

[6] Kemnitzer W, Drewe J, Jiang S (2007). Discovery of 4-aryl-4H-chromenes as a new series of apoptosis inducers using a cell- and caspase-based high-throughput screening assay. 3. Structure-activity relationships of fused rings at the 7,8-positions. J Med Chem.

[7] Schmitt F, Gold M, Rothemund M (2019). New naphthopyran analogues of LY290181 as potential tumor vascular-disrupting agents. Eur J Med Chem.

[8] Schmitt F, Schobert R, Biersack B (2019). New pyranoquinoline derivatives as vascular-disrupting anticancer agents. Med Chem Res.

[9] Dell CP, Singh JP, Smith CW

[10] Wood DL, Panda D, Wiernicki TR, Wilson L, Jordan MA, Singh JP (1997). Inhibition of mitosis and microtubule function through direct tubulin binding by a novel antiproliferative naphthopyran LY290181. Mol Pharmacol.

[11] Yusenko MV, Biyanee A, Frank D (2021). Bcr-TMP, a novel nanomolar-active compound that exhibits both MYB- and microtubule-inhibitory activity. Cancers (Basel).

[12] Cicirò Y, Sala A (2021). MYB oncoproteins: emerging players and potential therapeutic targets in human cancer. Oncogenesis.

[13] Köhler LHF, Reich S, Begemann G, Schobert R, Biersack B (2022). 2-Amino-4-aryl-5-oxo-4,5-dihydropyrano[3,2-c]chromene-3-carbonitriles with microtubule-disruptive, centrosome-declustering, and antiangiogenic effects *in vitro* and *in vitro*. ChemMedChem.

[14] Cai SX, Zhang H, Jiang S, Storer R

[15] Grossman D, Altieri DC (2001). Drug resistance in melanoma: mechanisms, apoptosis, and new potential therapeutic targets. Cancer Metast Rev.

[16] Lawson ND, Weinstein BM (2002). In vivo imaging of embryonic vascular development using transgenic zebrafish. Dev Biol.

[17] Chayka O, Kintscher J, Braas D, Klempnauer KH (2005). v-Myb mediates cooperation of a cell-specific enhancer with the mim-1 promoter. Mol Cell Biol.

[18] Yusenko M, Jakobs A, Klempnauer KH (2018). A novel cell-based screening assay for small-molecule MYB inhibitors identifies podophyllotoxins teniposide and etoposide as inhibitors of MYB activity. Sci Rep.

[19] Lau VM, Pfalzgraff WC, Markland TE, Kanan MW (2017). Electrostatic control of regioselectivity in Au(I)-catalyzed hydroarylation. J Am Chem Soc.

[20] Köhler LHF, Reich S, Yusenko M (2022). A new naphthopyran derivative combines c-myb inhibition, microtubule-targeting effects, and antiangiogenic properties. ACS Med Chem Lett.

[21] Verschraagen M, Koks CH, Schellens JH, Beijnen JH (1999). P-glycoprotein system as a determinant of drug interactions: the case of digoxin-verapamil. Pharmacol Res.

[22] Biersack B, Muthukumar Y, Schobert R, Sasse F (2011). Cytotoxic and antivascular 1-methyl-4-(3-fluoro-4-methoxyphenyl)-5-(halophenyl)-imidazoles. Bioorg Med Chem Lett.

[23] Schwartz EL (2009). Antivascular actions of microtubule-binding drugs. Clin Cancer Res.

[24] Arnaoutova I, Kleinman HK (2010). In vitro angiogenesis: endothelial cell tube formation on gelled basement membrane extract. Nat Protoc.

[25] Cross LM, Cook MA, Lin S, Chen J, Rubinstein AL (2003). Rapid analysis of angiogenesis drugs in a live fluorescent zebrafish assay. Arterioscler Thromb Vasc Biol.

[26] Choueiri TK (2008). Axitinib, a novel anti-angiogenic drug with promising activity in various solid tumors. Curr Opin Pharmacol.

[27] Gao X, Aguanno D, Board M, Callaghan R (2021). Exploiting the metabolic energy demands of drug efflux pumps provides a strategy to overcome multidrug resistance in cancer. Biochim Biophys Acta Gen Subj.

[28] Yanase K, Tsukahara S, Asada S, Ishikawa E, Imai Y, Sugimoto Y (2004). Gefitinib reverses breast cancer resistance protein-mediated drug resistance. Mol Cancer Ther.

[29] Leggas M, Panetta JC, Zhuang Y (2006). Gefitinib modulates the function of multiple ATP-binding cassette transporters *in vivo*. Cancer Res.

[30] Sharom FJ (2011). The P-glycoprotein multidrug transporter. Essays Biochem.

[31] Scala S, Akhmed N, Rao US (1997). P-glycoprotein substrates and antagonists cluster into two distinct groups. Mol Pharmacol.

[32] Lei ZN, Teng QX, Wu ZX (2021). Overcoming multidrug resistance by knockout of ABCB1 gene using CRISPR/Cas9 system in SW620/Ad300 colorectal cancer cells. MedComm.

[33] Su M, Huang J, Liu S (2016). The anti-angiogenic effect and novel mechanisms of action of Combretastatin A-4. Sci Rep.

[34] Anfossi G, Gewirtz AM, Calabretta B (1989). An oligomer complementary to c-myb-encoded mRNA inhibits proliferation of human myeloid leukemia cell lines. Proc Natl Acad Sci USA.

[35] Calabretta B, Sims RB, Valtieri M (1991). Normal and leukemic hematopoietic cells manifest differential sensitivity to inhibitory effects of c-myb antisense oligodeoxynucleotides: an *in vitro* study relevant to bone marrow purging. Proc Natl Acad Sci USA.

[36] Guérin M, Zheng ZM, Andrieu N, Riou G (1990). Strong association between c-myb and oestrogen-receptor expression in human breast cancer. Oncogene.

[37] Drabsch Y, Hugo H, Zhang R (2007). Mechanism of and requirement for estrogen-regulated MYB expression in estrogen-receptor-positive breast cancer cells. Proc Natl Acad Sci USA.

[38] Biroccio A, Benassi B, D’agnano I (2001). c-Myb and Bcl-x overexpression predicts poor prognosis in colorectal cancer. Am J Pathol.

[39] Hugo H, Cures A, Suraweera N (2006). Mutations in the MYB intron I regulatory sequence increase transcription in colon cancers. Gene Chromosome Canc.

[40] Degirmenci U, Yap J, Sim YRM, Qin S, Hu J (2021). Drug resistance in targeted cancer therapies with RAF inhibitors. Cancer Drug Resist.

[41] Li Y, Huang Y, Cheng H (2022). Discovery of BRAF/HDAC dual inhibitors suppressing proliferation of human colorectal cancer cells. Front Chem.

